# Neutralizing Antibodies against SARS-CoV-2 Beta and Omicron Variants Inhibition Comparison after BNT162b2 mRNA Booster Doses with a New PETIA sVNT Assay

**DOI:** 10.3390/diagnostics13050889

**Published:** 2023-02-26

**Authors:** Marta Fogolari, Bruno Daniele Leoni, Marina De Cesaris, Rita Italiano, Flavio Davini, Ginevra Azzurra Miccoli, Daniele Donati, Luigi Clerico, Andrea Stanziale, Giovanni Savini, Nicola Petrosillo, Massimo Ciccozzi, Lorenzo Sommella, Elisabetta Riva, Paolo Fazii, Silvia Angeletti

**Affiliations:** 1 Clinical Laboratory Unit, Fondazione Policlinico Universitario Campus Bio-Medico, 00128 Rome, Italy; 2 Unit of Clinical Laboratory Science, Department of Medicine and Surgery, University Campus Bio-Medico, 00128 Rome, Italy; 3 Abbott, Core Diagnostics, 00144 Rome, Italy; 4 Infection Prevention and Control Service, Fondazione Policlinico Universitario Campus Bio-Medico, 00128 Rome, Italy; 5 Clinical Microbiology and Virology, Spirito Santo Hospital, 65122 Pescara, Italy; 6 Istituto Zooprofilattico Sperimentale dell’Abruzzo e del Molise ‘G Caporale’, 64100 Teramo, Italy; 7 Unit of Medical Statistics and Molecular Epidemiology, University Campus Bio-Medico of Rome, 00128 Rome, Italy; 8 Health Management, Fondazione Policlinico Universitario Campus Bio-Medico, 00128 Rome, Italy; 9 Unit of Virology, University Campus Bio-Medico of Rome, 00128 Rome, Italy

**Keywords:** SARS-CoV-2, VOCs, serum antibodies, vaccine, neutralization assay, PETIA assay

## Abstract

Background: Monitoring antibody response following SARS-CoV-2 vaccination is strategic, and neutralizing antibodies represent the gold standard. The neutralizing response to Beta and Omicron VOCs was evaluated versus the gold standard by a new commercial automated assay. Methods: Serum samples from 100 healthcare workers from the Fondazione Policlinico Universitario Campus Biomedico and the Pescara Hospital were collected. IgG levels were determined by chemiluminescent immunoassay (Abbott Laboratories, Wiesbaden, Germany) and serum neutralization assay as the gold standard. Moreover, a new commercial immunoassay, the PETIA test Nab (SGM, Rome, Italy), was used for neutralization evaluation. Statistical analysis was performed with R software, version 3.6.0. Results: Anti-SARS-CoV-2 IgG titers decayed during the first ninety days after the vaccine second dose. The following booster dose significantly (*p* < 0.001) increased IgG levels. A correlation between IgG expression and neutralizing activity modulation was found with a significant increase after the second and the third booster dose (*p* < 0.05. Compared to the Beta variant of the virus, the Omicron VOC was associated with a significantly larger quantity of IgG antibodies needed to achieve the same degree of neutralization. The best Nab test cutoff for high neutralization titer (≥1:80) was set for both Beta and Omicron variants. Conclusion: This study correlates vaccine-induced IgG expression and neutralizing activity using a new PETIA assay, suggesting its usefulness for SARS-CoV2 infection management.

## 1. Introduction

The SARS-CoV-2 virus is still spreading worldwide, with more than 650 million cases. Five vaccines have been approved in Italy to mitigate virus spread: Comirnaty (Pfizer/BioNTech), Spikevax (Moderna), Vaxzevria (AstraZeneca), Ad26.COV2. S (Johnson & Johnson) and Nuvaxovid (Novavax). Following the Italian government regulations, healthcare workers (HCWs) were among the first groups to receive the vaccination. The vaccine used was Pfizer BNT162b2, a nucleoside-modified messenger RNA (mRNA) encoding the viral spike (S) glycoprotein of SARS-CoV-2 [1]. As for other public vaccination programs, monitoring antibody response following vaccination was not considered relevant in the cost/benefit analysis, despite much scientific literature suggesting that serological assays can provide important information on vaccination efficacy [2,3]. In particular, several authors have indicated that anti-RBD antibodies can be strongly associated with neutralizing activity through ACE2/RBD binding inhibition [4,5,6,7]. More recently, infections have been correlated with low levels of neutralizing antibodies [8]. Neutralizing antibody measuring assays are considered the gold standard for evaluating the protective immune response of an organism. However, they require live virus or pseudo-virus and a high level of biosecurity to be performed. In addition, they are often demanding and mostly impractical when dealing with many samples [9]. For this reason, commercial neutralization assays have been developed mainly as enzyme-linked immunoassays (ELISA) [10,11,12]. Nevertheless, while ELISA is a commonly used method, it has some disadvantages, such as the requirement of multiple washing steps, which can be time-consuming and difficult to automate. On the other hand, particle-enhanced turbidimetric inhibition assay (PETIA), does not require washing steps, even while maintaining high sensitivity. This results in a faster and more suitable tool for high-throughput screening that is efficient to perform in a laboratory setting [13].

In this context, these assays simulate in vitro the process through which neutralizing antibodies block the interaction between the viral RBD and the ACE2 receptor by binding the RBD protein of SARS-CoV-2 in a subject exposed to the virus.

Most of these assays have been developed against the receptor-binding domain of the Wuhan original variant.

The spreading of new virus variants has determined multiple mutations in this site, which might have affected the validity and the neutralization capability of these immunoassays for detecting anti-SARS-CoV-2 spike and RBD antibodies [14]. However, several studies have shown that although there is a reduced efficiency, a booster dose was still able to stimulate anti-RBD antibodies correlated with neutralizing and protective activity even against Omicron, the latest variant of concern (VOC) [15,16].

In this paper, we have evaluated and compared the neutralizing response to the B.1.351 (Beta) and B.1.1.529 (Omicron) VOCs induced by Pfizer vaccination booster doses through a new commercial automated CE-marked immunoassay, and compared it to the serum neutralization assay standard reference test.

## 2. Materials and Methods

### 2.1. Human Samples

Serum samples of 100 healthcare workers of the Campus Biomedico (Rome, Italy) and the Pescara Hospital were obtained between February and June 2021 and January 2022, respectively. Samples were collected before and twenty-one days after Pfizer Comirnaty’s third dose administration and from 21 to 90 days after the second booster dose. They were centrifuged, frozen and stored at −80 °C until testing, per internal procedures. The patients were constantly monitored for the lack of previous and contextual infection. Written informed consent was obtained from all participating individuals in accordance with the local ethical committee requirements (IRB number 8.1(21) OSS).

### 2.2. Serological Assays

Anti-SARS-CoV-2 RBD IgG levels have been determined by an automated chemiluminescent immunoassay (Alinity anti-SARS-CoV-2 IgG II Quantitative, Abbott Laboratories, Wiesbaden, Germany). The results are expressed per the WHO standard preparation for SARS-CoV-2 binding antibodies alignment (1 BAU/mL = 0.142 × AU/mL). The assay linearity of the method has been previously assessed in independent evaluations [17] and precision was verified in the assay insert expected ranges.

SARS-CoV-2 serum neutralization (SN) assay was performed at the Istituto Zooprofilattico Sperimentale dell’Abruzzo e Molise (Teramo, Italy, IZSTe) under biosafety level 3 (BSL-3) conditions. The neutralization titer was defined as the reciprocal of the highest serum dilution without any cytopathic effect (CPE) in the wells. The positivity threshold was set at 1:10. Positive and negative control sera, kindly provided by the Istituto Nazionale Malattie Infective “Lazzaro Spallanzani” (INMI, Rome, Italy), were included in each run. For this study, a 1:80 dilution titer was considered a robust neutralizing capacity threshold in line with the SN method specification. Neutralization capabilities of vaccine-induced antibodies were measured versus the B.1.351 (Beta) and B.1.1.529 (Omicron) VOCs, and the viral titer of the stocks was determined by TCID50 assay as previously described [18]. Due to test availability issues, patient serum samples were examined only before and after the third booster dose.

A new commercial Particle Enhanced Turbidimetric Immunoassay (PETIA) test named Nab (SARS-CoV-2 Neutralizing Antibodies REF 8003; SGM, Rome, Italy) was applied to the Abbott Alinity c instrument (Abbott Laboratories, Chicago, IL, USA). Briefly, the automated Nab assay simulates in vitro the process in which neutralizing antibodies block the interaction between the viral native RBD domain and the ACE2 receptors by binding the RBD protein of SARS-CoV-2. The presence of serum anti-RBD neutralizing antibodies competes with reagent assay recombinant RBD-ACE2 antigen binding. The manufacturer validated this assay in comparison with TCID_50_ as a reference gold standard method indicating the following interpretative cutoffs for neutralization inhibition rate (%): absent (≤25%); moderate (25–56%); high (>56%). The latter threshold corresponded to the TCID_50_ dilution rate related to the high neutralization titer. The assay was calibrated to return the inhibition rate (%) and controlled with dedicated quality controls. The higher the concentration of neutralizing antibodies, the higher the expected inhibition rate. The obtained value was validated and confirmed by absorbance retrieved values according to the following formula: Inhibition rate (%) = [1 − (OD548nm sample/OD548 nm total negative control)] × 100. The assay precision assessment was performed on the Nab assay per Clinical Laboratory Standards Institute (CLSI) EP15A3 protocols using two quality control levels for five consecutive days, five times a day, and found not exceeding a CV of 3.2% (data not shown). The assay linearity was verified in line with the manufacturer’s statements per the CLSI EP6 protocol. Briefly, intermediate samples were prepared by mixing a high sample with a blank sample. Successive dilution and linearity were assessed versus dilution-expected concentrations.

### 2.3. Statistics

The statistical analysis results are presented as mean ± standard deviation (SD) for continuous variables, medians with Q1 and Q3 when variables were non-normally distributed, and as a percent (%) for categorical variables. Differences in categorical variables between groups were evaluated with Pearson’s χ^2^ or Fisher’s exact test. Differences in customarily distributed populations were assessed with ANOVA. Distribution normality was tested with the Shapiro–Wilk test. Data distribution was represented in box-and-whiskers (Boxplot) format. The band within the box represents the median, lower and upper borders denote the 25th and 75th percentile of the distribution, respectively, and the whiskers represent the minimum and maximum of Tukey’s fences values. The correlation was evaluated by the Spearman method. Receiver operating characteristic (ROC) curve analysis was applied for cutoff estimation and specificity/sensitivity evaluation at different thresholds. ROC curve AUC comparison was performed with the Delong method. Multiple comparisons in non-normal distribution were performed with the Steel–Dwass–Critchlow–Fligner pairwise ranking non-parametric method. ROC curves were developed to investigate sensitivity and specificity. Concordance was assessed with Cohen kappa statistics. A *p*-value of less than 0.05 was considered statistically significant for all experiments, and the sample size was chosen for having a statistical power of 0.8. Data are reported at the 95% Confidence Interval. The dataset was built in Microsoft Excel (Microsoft Corporation, Redmond, WA, USA), and statistical analysis was performed with Analyze-it for Microsoft Excel 4.92.4 and R software version 3.6.0 (https://www.R-project.org/, accessed on 26 April 2019).

## 3. Results

The age of the healthcare workers participating in this study was 43.5 ± 13 years, with a female/male ratio of 61%. The first group’s (age = 29.0 ± 7 years, female ratio 70%; *n* = 40) immune response was evaluated from twenty-one to ninety days following the Pfizer Comirnaty second dose booster. During the first ninety days, the anti-SARS-CoV-2 IgG titers decayed from 2960 to 652 BAU/mL. In the second group (53.0 ± 13 years, female ratio 55%, *n* = 60), immune response was evaluated suddenly before and twenty-one days after the third dose booster. The first sampling of this group was nine months after the second dose. As expected, the booster dose significantly (*p* < 0.001) increased specific anti-IgG levels (Table 1). The median levels increased significantly from 97 to 3453 BAU/mL (*p* < 0.001). All the examined healthcare workers had moderate to high immune responses.

The antibody neutralizing activity was also investigated using the commercial PETIA sVNT (Nab) method. As shown in Table 1, there is a correlation between anti-IgG expression and neutralizing activity modulation. As expected, the average of neutralizing antibodies (Nabs) inhibition rates (%) significantly increased following the second booster dose (*p* < 0.05). Neutralizing-antibody-mediated inhibition slowly decayed in a time-dependent fashion from 89.05 ± 5.29% to 70.75 ±16.23% in the first ninety days and down to 37.48 ± 11.67% after nine months. The efficiency of neutralizing antibodies was fully restored (95.98%) twenty-one days after the third booster dose.

**Figure 1:**
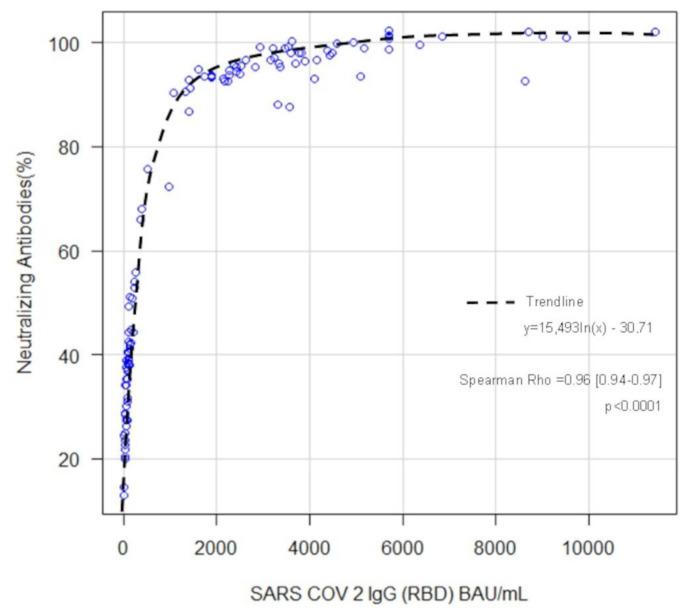
Anti-SARS-CoV-2- IgG and Neutralizing antibodies (Nab) correlation. Anti-RBD IgG levels are plotted against neutralizing-antibody-mediated percentage inhibition. The trend line equation is depicted in the picture: Nab% inhibition = 15,493 ln(x) − 30.71. Spearman Rho correlation was 0.96 (*p* < 0.001). Blue circles correspond to single value determination.

Figure 1 shows a non-linear regression depicting the neutralization inhibition rate against SARS-CoV-2 IgG concentration independently from the dose administration. We found a positive significant (Spearman Rho = 0.96) logarithmic correlation described by the following function Nabs% = 15,493 ln(SARS-CoV-2 IgG) − 30.71.

**Figure 2:**
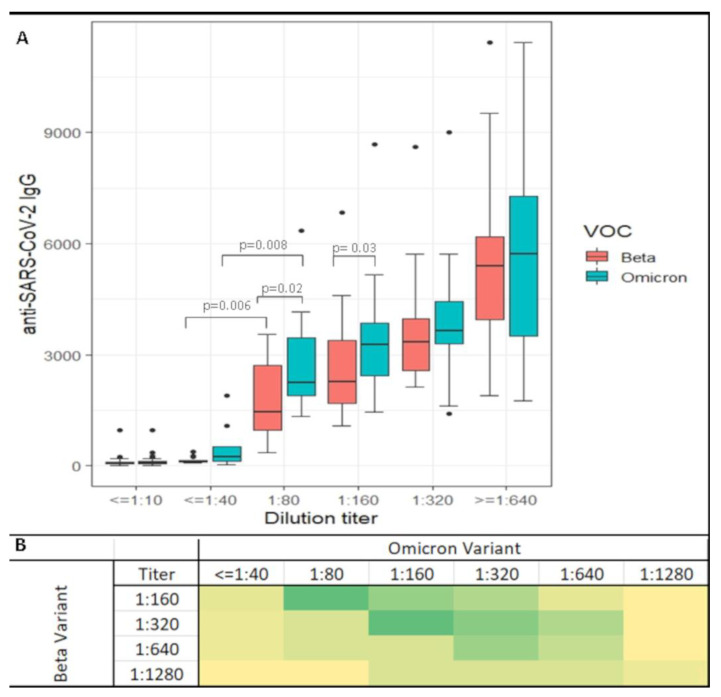
Box-and-whiskers plot of anti-SARS-CoV-2 IgG receptor-binding domain (RBD) levels at different neutralization. Dilution titers before and after Comirnaty vaccine third dose administration. Box plots indicate the median and interquartile range (IQR); the whiskers represent 1.5 times the IQR. Significant *p*-values are indicated over the brackets. Colors are related to the analyzed VOC as depicted in the picture legend (Panel (**A**)). Titer distribution comparison of Beta and Omicron VOCs after the third dose. Color intensity shading from light yellow to green reflects the number of subjects associated with the different variant dilution titers (panel (**B**)).

Neutralizing antibody levels were evaluated with the serum neutralization assay. A dilution titer was calculated for each sample measuring Beta and Omicron variant neutralizing responses in sixty patients before and after the third booster dose. The SARS-CoV-2 virus variant inhibition rates were compared to the anti-SARS-CoV-2 IgG levels. Data in Figure 2A show that both VOCs maintained the correlation between SARS-CoV-2 IgG (RBD) levels and the neutralizing titers. However, at the same antibody titer, different neutralizing ratios were seen. Indeed, the Omicron variant is associated with a significantly higher amount of antibodies corresponding to the same neutralization level. High neutralization titer (>1:80) was reached at a median of 1800 BAU/mL for Beta and 2400 BAU/mL (*p* < 0.001) for the Omicron variant. This difference remained even at a higher dilution. A matrix heatmap comparing the same subject titer readings for the virus variants after booster dose induction is shown in Figure 2B. Of the sixty patients examined, four did not show Omicron neutralization. On the other hand, while the booster dose enhanced substantial neutralizing activity in both variants, differences were recorded. Indeed, most patients had a lower response to Omicron compared to Beta. In particular, subjects neutralizing the Beta variant at 1:320 titer showed a titer of 1:160 or less to neutralize Omicron, while the antibodies neutralizing the Beta variant titered at 1:160 were less efficient with Omicron (1:80 or less). Nevertheless, the trend was inverted in a few subjects, where the third-dose-induced antibodies better neutralized Omicron than the Beta VOC.

We calculated the best Nab cutoff for high neutralization titer (≥1:80) in both variants. ROC curves were developed, and areas under the curves (AUC) were 0.99 (CI: 0.99–1.00) for the Beta variant and 0.98 (CI: 0.96–1.00) for the Omicron variant. The calculated Nab% cutoffs by the Youden index were 72% and 86% for Beta and Omicron VOCs, respectively. ROC curves were also developed on the whole population for anti-SARS-CoV-2 IgG at high neutralization dilution titer (>1:80 as per MNT IC90). In this case, areas under the curves (AUC) were calculated at 0.97 (CI95%: 0.95–0.99) for the Beta variant and 0.95 (CI95%: 0.92–0.97) for the Omicron variant. The anti-SARS-CoV-2 IgG cutoff by the Youden index was recalculated considering the above retrieved high neutralization thresholds, and measured at 597 and 1018 BAU/mL for Beta and Omicron, respectively. The specificity and sensitivity were evaluated in correspondence to the Nab high neutralization threshold indicated by the test manufacturer (Criterion => 56%) or Nab threshold derived from MNT comparison for both considered variants of concern. In this case, while for the Beta variant, the specificity (1.00) and sensitivity (0.97) were still reasonable, the Omicron-related data showed a significant decrease in specificity values (0.60) (Table 2).

## 4. Discussion

Vaccines were developed to produce a polyclonal immune response against the wild-type SARS-CoV-2 strain [19]. Several studies have shown that vaccination-induced immune response produced neutralizing antibody patterns cross-reacting with VOCs to gain protection against symptomatic reinfection [20,21,22]. Nevertheless, contradictory data have been described for Omicron about vaccine-induced neutralization capability [23,24,25].

In our study, we investigated neutralizing antibodies in a BNT162b2-vaccinated health worker population dynamically represented; 40 subjects were followed for up to 90 days after the second dose, and a second cluster (60 Subjects) was measured before and after the Pfizer vaccine third booster dose inoculation. As expected, the anti-RBD IgG antibody level decrease was time-dependent. According to what was described elsewhere [26], the decay score was constant, with antibody titer continuously decreasing about every 32 days during the considered period (data not shown). Neutralizing activity was measured simultaneously and reported as an inhibition rate (%). The highest neutralizing activities were associated with the highest antibody concentrations. However, the neutralization rate slowly waned after the second dose, was lost entirely after nine months and restored after a new booster dose inoculation. Recent publications showed a significant correlation between anti-SARS-Co-V 2 IgG (RBD) and neutralization activity measured with sVNT and in vitro SN assay [27,28]. In our case, the correlation was calculated as exponentially positive (Rho = 0.90) (*p* ≤ 0.001) and represented by the mathematical equation NAbs% = 15,493*ln (Anti RBD IgG) − 30.71. We further investigated neutralization titers using SN assay for Beta and Omicron VOCs. Although the correlation between anti-RBD IgG and neutralization level was maintained for both VOCs, the Omicron variant infection was, on average, less neutralized at the same level of anti-SARS-CoV-2 IgG (RBD). These results are in line with recent evidence about Omicron-induced immune response [29,30].

Moreover, this evidence adds insight to the discussion that the presence of antibodies does not necessarily imply an efficient neutralization [31]. It appears that the infecting variant could affect the antibody thresholds necessary to obtain a specific neutralizing activity, and that correlates of protection thresholds are unlikely to be universal. In this context, since the PETIA sVNT Nab test manufacturer indicated that a 56% inhibition rate limit was related to high neutralizing activity in accordance with the response to the original strain of SARS-CoV-2 virus, we evaluated if the proposed threshold was applicable to the Beta and Omicron variants as well. Indeed, the Omicron variant of SARS-CoV-2 has raised significant concerns due to its high number of mutations in the spike protein, including in the receptor binding domain (RBD) [32] These mutations may affect the ability of neutralizing antibodies, which could impact the virus’s transmissibility, pathogenicity and vaccine efficacy. Recent studies have shown that the Omicron variant can evade immunity from previous SARS-CoV-2 infections or vaccination, reducing the efficacy of anti-SARS-CoV-2 IgG antibodies and viral neutralization. In particular, Cele et al. found that the neutralization of Omicron was significantly reduced (22-fold decrease) compared to the ancestral virus in both infected and vaccinated individuals [33].

In line with these observations, our results show that a far higher threshold (87%) was needed for Omicron VOC compared to Beta (72%) and the original strain (56%). Nevertheless, since the thresholds were all included in the test linearity, the sVNT Nab test was still functional.

ROC curves were developed based on the whole population. High neutralization thresholds at the best sensitivity and specificity were recalculated. Our data indicated no statistically significant differences in retrieved cutoff for wild-type (352 BAU/mL) and Beta variant strains (597 BAU/mL).

Interestingly, this limit aligns with the anti-RBD IgG correlates of protection suggested by Feng et al. (506 BAU/mL) [13]. Since these values have been suggested as the reference cutoff for vaccination efficacy against symptomatic COVID-19, even in immunosuppressed people [34], this finding stimulates discussion about a possible protective threshold shift when dealing with different variants. In this context, our data showed a higher and more significant threshold for the Omicron Variant (1080 BAU/mL). We have no direct evidence that these retrieved thresholds can be protective against infection. However, a recent paper by H.J. Zar et al. reported similar values were associated with the capability for inhibition of symptomatic reinfection of 50% by the SARS-CoV-2 Omicron variant in vaccinated people (868 BAU/mL). These limits were consistently higher than those found in previously considered VOCs [35].

In addition, although in our experience, the Pfizer BNT162b2 booster third dose has stimulated the production of a higher quantity of antibodies compared to the second dose, the evidence that a much higher level of anti-SARS-CoV-2 IgG antibodies is needed to achieve the same level of neutralization raises questions about the duration of vaccine-induced immunity in the current variant of the virus. Accordingly, recent studies have shown that with Omicron, the neutralizing antibody titers after booster doses of both BNT162b2 and mRNA-1273 (Moderna) vaccines seem to decline more rapidly, reducing vaccine efficacy to 90 days and impacting clinical decisions, revaccination time tables for patients and health workers and supporting the importance of formulating updated vaccines against circulating SARS-CoV-2 variants [36].

We did not consider anti-SARS-CoV-2 RBD IgG levels and neutralization response in non-vaccinated, immunosuppressed or reinfected healthcare workers; this can be considered a study limitation.

## 5. Conclusions

In conclusion, even if further studies are required, this is the first paper correlating vaccine-induced anti-RBD IgG expression and neutralizing activity for the Beta and Omicron VOCs by using a new PETIA test on a high-throughput platform in comparison with the SN gold standard assay. Several authors indicated that sVNT limitations are due to a lack of standardization and valuable information on the variant response [37]. This study, built to evaluate a new high throughput neutralization test in a large population compared to actual reference methods, demonstrated that sVNT has an intrinsic value for measuring the variant of concern impact over vaccines constructed towards the same epitope. Consequently, introducing new vaccines aimed at producing specific anti-VOC antibodies will necessarily imply a resetting of the assay to have a meter aligned with the current vaccine and be able to measure its neutralizing capacity and the deviation caused by future VOCs. In addition, since neutralizing antibodies are a consequence of IgG expression after vaccination and the immune response to vaccination can vary in different subjects [38], we propose that a combined serological approach, which correlates SARS-CoV-2 IgG levels and neutralizing activity with the current variant of concern, could be helpful. It provides insights into single-subject antibody expression and the decline of the neutralization titers with time, which could help monitor vaccination efficacy and clinician decisional workflows for revaccination in exposed subjects.

## Figures and Tables

**Table 1:** Population Characteristics. Abbreviations: IQR = interquartile range; SD = Standard deviation; Nab = Neutralizing antibodies; *N* = Number of subjects; 21d = twenty-one days, 90d = ninety days, 9m = nine months.

			Group 1		Group 2		
*N*		100	40		60		
Age average ± SD		43.5 ± 13	29.0 ± 7		53.0 ± 11		
Gender *N* (%)	Female	61 (61)	28 (70)		33 (55)		
	Male	39 (39)	12 (30)		27 (45)		
			**2nd dose**			**3rd dose**	***p*** **-value**
			**21d**	**90d**	**9m**	**21d**	
SARS-CoV-2 IgG (Median [IQR])			2.960 [2040–4654]	652 [337–1023]	97 [59–123]	3.453 [2283–4945]	<0.001
NAb% (Average ± SD)			89.05 ± 5.29	70.75 ±16.23	37.48 ± 11.67	95.98 ± 4.56	<0.001

**Table 2:** Variant-related comparison of Receiver-Operating Characteristics (ROC) curves for anti-SARS-CoV-2 IgG receptor-binding domain (RBD) values (BAU/mL) and neutralizing antibody percent inhibition rate (Nab%) at 1:80 neutralization dilution titer in vaccinated subjects. The area under the curve (AUC), fitting p-value and calculated specificity and sensitivity are included in the picture. The optimal cutoff point (Youden Threshold) was defined as the minimum value of (1-sensitivity)^2^ + (1-specificity)^2^. Chosen thresholds are referred to as “Criterion”.

TEST	VOC	AUC [IC95]	*p*-Value	Youden Threshold	Criterion	Sensitivity [IC95]	Specificity [IC95]	MNT Agreement Kappa [IC95]
Nab%	Beta	0.99 [0.99–1.00]	0.98	72	-	0.98 [0.91–0.99]	0.98 [0.91–0.95]	1.00 [0.98–1.00]
				-	56	1.00 [0.94–1.00]	0.96 [0.88–0.99]	1.00 [0.98–1.00]
	Omicron	0.98 [0.96–1.00]	0.02	87	-	1.00 [0.94–1.00]	0.95 [0.87–0.98]	1.00 [0.98–1.00]
				-	56	1.00 [0.93–1.00]	0.87 [0.77–0.93]	1.00 [0.98–1.00]
SARS-CoV-2 IgG BAU/mL	Wt Strain Threshold (56%)	0.99 [0.98–1.00]	-	352	-	0.98 [0.91–0.99]	0.99 [0.91–0.99]	0.97 [0.92–1.00]
	Beta Threshold (72%)	0.97 [0.95–0.99]	0.94	597	-	0.95 [0.94–0.99]	0.98 [0.75–0.86]	0.97 [0.92–1.00]
				-	352	0.99 [0.97–1.00]	0.80 [0.75–0.90]	0.87 [0.78–0.96]
	Omicron Threshold (87%)	0.95 [0.92–0.97]	0.03	1018	-	0.95 [0.97–1.00]	1.00 [0.93–0.98]	0.95 [0.89–1.00]
				-	352	1.00 [0.97–1.00]	0.60 [0.52–0.68]	0.87 [0.78–0.96]

## Data Availability

Data are available if requested.
